# High performance of targeted next generation sequencing on variance detection in clinical tumor specimens in comparison with current conventional methods

**DOI:** 10.1186/s13046-017-0591-4

**Published:** 2017-09-07

**Authors:** Dan Su, Dadong Zhang, Kaiyan Chen, Jing Lu, Junzhou Wu, Xinkai Cao, Lisha Ying, Qihuang Jin, Yizhou Ye, Zhenghua Xie, Lei Xiong, Weimin Mao, Fugen Li

**Affiliations:** 1 Pathology Department, Zhejiang Cancer Hospital, Hangzhou, 310022 China; 2 Key Laboratory of Diagnosis and Treatment Technology on Thoracic Oncology of Zhejiang Province, Hangzhou, 310022 China; 3 The Research and Development Center of Precision Medicine, 3D Medicine Inc., Shanghai, 201114 China; 4 Changhai Hospital, The Second Military Medical University, Shanghai, 200433 China

**Keywords:** Targeted next generation sequencing, Amplification-refractory mutation system, Fluorescence in situ hybridization, Immunohistochemistry, Clinical tumor samples

## Abstract

**Background:**

Next generation sequencing (NGS) is being increasingly applied for assisting cancer molecular diagnosis. However, it is still needed to validate NGS accuracy on detection of DNA alternations based on a large number of clinical samples, especially for DNA rearrangements and copy number variations (CNVs). This study is to set up basic parameters of targeted NGS for clinical diagnosis and to understand advantage of targeted NGS in comparison with the conventional methods of molecular diagnosis.

**Methods:**

Genomic DNA from 1000 Genomes Project and DNA from cancer cell lines have been used to establish the basic parameters for targeted NGS. The following confirmation was conducted by clinical samples. The multiple variants tested by amplification-refractory mutation system (ARMS), fluorescence in situ hybridization (FISH) and immunohistochemistry (IHC) were evaluated by targeted NGS to determine the sensitivity. Furthermore, the multiple variants detected by targeted NGS were confirmed by current conventional methods to elucidate the specificity.

**Results:**

At sequencing depth of 500×, the maximal sensitivities on detecting single nucletic variances (SNVs) and small insertions/deletions (Indels) can reach 99% and 98.7% respectively, and in 20% of cancer cells, CNV detection can reach to the maximal level. The following confirmation of the sensitivity and specificity was conducted by a large cohort of clinical samples. For SNV and indel detection in clinical samples, targeted NGS can identify all hotspot mutations with 100% sensitivity and specificity. On *ALK* fusion detection, about 86% IHC-identified cases could be identified by targeted NGS and all *ALK* fusion detected by targeted NGS were confirmed by IHC. For *HER2*-amplification, 14 *HER2*-amplification cases identified by target NGS were all confirmed by FISH and about 93.3% of Her-2 IHC (3+) cases were identified by targeted NGS. Finally, the targeted NGS platform developed here has accurately detected *EGFR* hotspot mutations in 215 NSCLC patients.

**Conclusions:**

DNA from cancer cell lines is better than standard DNA as a reference to establish basic parameters for targeted NGS. Comparison of the conventional methods using a large cohort of patient samples confirmed the high preformance of targeted NGS on detecting DNA alterations.

**Electronic supplementary material:**

The online version of this article (10.1186/s13046-017-0591-4) contains supplementary material, which is available to authorized users.

## Background

Cancer is considered to be caused by both inherited and acquired genomic alterations, which leads to uncontrolled cell growth. Over the past 20 years, new-drug development has focused on the known oncogenic drivers and heralded an era of targeted therapies. Compared to traditional cytotoxic chemotherapy, targeted therapies are safer, more efficacious and less side effects. [1] For example, it has been reported the efficacy of targeted drugs, herceptin to patients with *ERBB2*-amplified breast cancer and gefitinib and erlotinib to patients with mutated *EGFR* lung cancer, is better. So far, hundreds of targeted drugs have been developed or under development, targeting the corresponding genomic alterations, including site mutations, insertions and deletions (Indels), copy number variants (CNVs) and DNA rearrangements. Identification of these alterations in cancer patients is the first step to provides the targets for therapy. The cancer biology is complex. For instance, the patients with *EGFR* mutated lung cancer are benefit from erlotinib and gelfitinib, but if *EFGR* harboring T790 M mutation, then resistant to these drugs. [2, 3] These suggest it is important to fully understand the DNA alterations in cancer patients.

Currently, the conventional technologies for identifying the genomic alterations in patients include the amplification-refractory mutation system (ARMS), fluorescence in situ hybridization (FISH) and immunohistochemistry (IHC). All of these methods have both advantages and limitations in application. These methods are well installed and highly reliable, but they have a common shortness: each genomic alteration is analyzed in a specific assay. The sensitivity of ARMS to detect site mutation from genomic DNA can reache to 0.10%, [4] but the technology is only used to detect the known base substitutions or Indels. ARMS can also be used for gene fusion detection at mRNA level, but the good quality RNA could be limited from formalin fixed, paraffin-embedded (FFPE) tissues. FISH is used to detect DNA rearrangements and amplifications at genomic level. This method is relatively rapid, well-standardized, rather expensive method. However, FISH can not distinguish fusion variants. IHC mainly detected the changes of gene expression at the protein level, which usually resulted in by gene amplification or DNA rearrangement.

Next generation sequencing (NGS) is the most powerful tool to accurately detect most gene alterations on a massive scale, allowing interrogation of all genes or selected genes in a single assay. This technology requires low amounts of DNA, and has high sensitivity and specificity. [5] Moreover, cancers are frequently caused by alterations on multiple genes, which collaborate to promote tumor development. [6] A combination of drugs targeting the multiple alterations may be an approach to achieve the best therapeutic efficacy. [7] The conventional methods are impossible to massively screen cancer-related genes in a single assay. Therefore, NGS has been increasingly used in clinical diagnosis. However, we need throughly validate the sensitivity and accuracy of NGS to detect multiple types of DNA alterations from a large number of clinical specimens, which have been confirmed by the current clinical methods.

In this study, we developed and validated a panel for targeted NGS which is able to detect multiple types of genomic alterations in 365 genes commonly associated with cancers. A number of clinical samples were collected, including 131 specimens for base substitutions and Indels, 18 for *ALK* fusions, 86 for *HER2* amplifications. These 235 clinical tissues were used to throughly compare the results from targeted NGS and conventional methods, including ARMS, FISH or IHC, and explore the cause of disconcordance. Finally, the capability of targeted NGS on detecting multiple types of genomic alterations was performed in 215 non-small-cell lung cancer (NSCLC) samples. The results may guide the clinician to select the right method for the diagnosis based on the characteristics of each method and clinical needs.

## Methods

### Standard DNA, cell lines and clinical tumor specimens

#### Clinical cancer specimens

This study was approved by Ethics Committee of Zhejiang Province Cancer Hospital,China. We have enrolled 235 formalin fixed, paraffin-embedded (FFPE) tumour specimens from lung adenocarcinoma, colon and other types of cancers in this study (Additional file 1: Table S1). The specimens have been reviewed by a pathologist to make sure the content of cancer cells is > = 20%.

#### Standard DNA

For base substitution validation, purified DNA from 15 lymphoblastoid cell lines from the 1000 Genomes Project were purchased from the Coriell Institute (Additional file 2: Table S2).

#### Cell lines

For indel validation, 28 immortalized tumor cell lines (Additional file 3: Table S3) were purchased from American Type Culture Collection (ATCC) (http://www.atcc.org/). For copy number validation, HCC1143 matched tumor and normal cell lines were purchased from ATCC as either cell pellets or DNA.

### Targeted NGS

#### Pathological examinations of the clinical tumor specimens

4-μm paraffin sections out of the clinical tumor specimens were stained with hematoxylin and eosin (HE) for pathological review to determine that a sample has a volume of ≥1 mm^3^ and ≥20% tumor cells. If the percentage of tumor cells was ≤20%, a macro-dissection was used for enrichement of tumor cells.

#### DNA extraction

Paraffin in Formalin Fixed Paraffin Embedded (FFPE) sections and cores was removed by xylenes, followed by ethanol washing. Tissues were digested by proteinase K at 56 °C overnight and incubated at 90 °C for 5 min to reverse DNA crosslink. Genomic DNA was then extracted with QIAamp DNA FFPE Tissue Kit (Qiagen) and quantified by PicoGreen fluorescence assay (Invitrogen).

#### Construction of sequencing libraries

50–200 ng of DNAs were fragmented to around ~200 bp by sonication (Covaris), and constructed into the libraries with KAPA Hyper Prep Kit (Kapa Biosystems). [8]

#### Capture of the targeted DNAs and sequencing

The baits, a pool of 16,198 individually synthesized 5′-biotinylated 120 bp DNA oligonucleotides (Integrated DNA Technology), cover 4557 exons of 365 cancer-related genes, 47 introns of 25 genes frequently re-arranged in cancer (Additional file 4: Table S4). Intronic baits were filtered for repetitive elements as defined by the UCSC Genome RepeatMasker track. [9] The targeted regions were captured with the baits as described previously. [10] Briefly, a pool of indexed sequencing libraries, total 1000 ng, was lyophilized and resuspended in water, heated to denature and kept at 68 °C. Then the bait, Cot, salmon sperm and adaptor-specific blocker DNA were added in the pool. After incubation, the library-bait duplexes were captured with Dynabeads M270 Streptavidin (Invitrogen) and off-target parts in the libraries were washed off by SSC. The PCR master mix was added to directly amplify the captured libraries, and followed by purification with 1.8 × SPRI, quantification by Qubit 3.0 (Life Technologies) and determination of the DNA size on LabChip GX (Caliper). Libraries were adjusted to 1.05 nM and seqeuneced in next generation sequencing platform illumina Nextseq 500.

### Analysis of DNA alterations

#### Sequence data processing

Sequence data were mapped to the human genome (hg19) using BWA aligner v0.7.12. PCR duplicate read removal and sequence metric collection were done using Picard 1.130 (https://github.com/broadinstitute/picard/releases/tag/1.130) and Samtools 0.1.19. Variant calling was done only in the targeted genomic regions.

#### Base substitution analysis

We used a Bayesian methodology, which allows detection of novel somatic mutations at low mutation allele frequency (MAF) and increase sensitivity for calling mutations at hotspots through the incorporation of tissue-specific prior expectations. The total reads in the variant position could not be less than 30, and the maximum variant frequency of normal controls was 0.03. Final calls were cut off at MAF > 1% (MAF > 0.5% at hotspots) after filtering for strand bias.

#### Indel analysis

To detect Indels, de novo local assembly in each targeted exon was performed using the de Bruijn approach. Pindel version 0.2.5a7 (https://github.com/genome/pindel/releases/tag/v0.2.5a7) was used to detect indels in this research. Filtering of Indel candidates was carried out as described for base substitutions above (strand bias >0.9 or <0.1, MAF threshold <1% while MAF < 0.5% at hotspots), with an empirically increased requirement at repeats.

#### CNA analysis

A statistically rigorous and computationally efficient algorithm called BIC-seq was used for detecting CNVs. In this algorithm, a poisson or other parametric models are not assumed on the read distribution as is done in other currently available methods, and it is thus more robust to outliers and datasets that cannot be well approximated with a parametric model. It is also fast and able to handle high-coverage genomes effectively. Furthermore, the statistical framework behind BIC-seq can be extended to the problem of identifying recurrent CNVs in multiple cancer genomes. We obtained a log-ratio profile of the sample by normalizing the sequence coverage obtained at all exons against a process-matched normal control. This profile was corrected for GC-bias.

#### DNA arrangement analysis

Genomic rearrangements were identified by analyzing the clipped reads which can be extracted by the tag information of bam files mapped by bwa software. Then candidate reads which are discordant or with the same direction are performed to be filtered. Read pairs for which reads mapped to separate chromosomes, or at a distance of over 2 kb are kept for fusion detection in probe level. Output rearrangements contain translocation, inversion, long deletion, etc.

### Amplification-refractory mutation system (ARMS) PCR

Mutational analyses of the *EGFR*, *KRAS*, *NRAS* and *BRAF* in 34 FFPE samples were carried out by ADx-ARMS Test Kits (Xiamen AmoyDx Biomedical Technology Co., Ltd.) in Zhejiang Province Cancer Hospital. Mutational analyses of the *EGFR* and *KRA*S in 97 FFPE samples from lung adenocarcinomas and colorectal cancers were carried out according to the ARMS method using Human *EGFR* Gene Twenty-nine Mutations Detection Kit and Human *KRAS* Gene Seven Mutations Detection Kit (PCR fluorescence probe method) (Wuhan YouZhiYou Medical Technology Co., Ltd.). Some *EGFR* mutations were confirmed by Applied Biosystems® 7500 Real-Time PCR Systems. After the reaction, the fluorescent signal curves and the threshold line were used to interpret the mutation results.

### Immunohistochemistry (IHC)

IHC was carried out using established methods. [11] In brief, sections were deparaffinized and incubated with the ALK work fluid (ALK IHC-5A4, Leica Biosystems) and ERBB2 work fluid (Her-2 IHC-UMAB36, ZSGB-BIO). A three-stage indirect immunoperoxidase technique was performed on a Benchmark Ventana staining module (Ventana, Tucson, AZ). Antigen retrieving was performed on the module using the cell conditioning buffer (CC1) pH 8.4 with Tris/Borate/EDTA (Ventana), for 1 h with the Amplification Kit (Ventana). The percentage of positive cells was evaluated, and staining scores were assessed as follows: 0, no staining; 1+, faint cytoplasmic staining; 2+, moderate cytoplasmic staining; and 3+, intense granular cytoplasmic staining.

### Fluorescence in situ hybridization (FISH)

FISH was carried out using established methods. [11] Briefly, FFPE tissue sections were hybridized with probes to *ERBB2* and the centromere region of chromosome 17 (*CEP17*) in the PathVysion *ERBB2* FISH assay (Abbott-Vysis). Hybridized slides were digitally imaged and 20 no overlapping cells were evaluated for *ERBB2* and *CEP17* copy numbers using the Ikoniscope *ERBB2* Analysis Software. *ERBB2* copy number, *CEP17* copy number and *ERBB2*:*CEP17* ratio were calculated and reported according to the package insert.

*ALK* rearrangement status was assessed by FISH using the Vysis *ALK* Break Apart FISH Probe Kit, and tests were performed according to the kit instruction. In brief, slides were baked for one hour at 60 °C followed by deparaffinization and rehydration. Pretreatment was performed at 80 °C for 20 min, followed by protease treatment for 22 min at 37 °C. The slides were dehydrated at 73 °C for 3 min and incubated with probes at 37 °C overnight. After washed at 75 °C for 3 min, the slides were mounted with 4′,6-diamidino-2-phenylindole (DAPI) (ProLong Gold Antifade Mountant with DAPI, ThermoFisher Scientific), and analyzed under a ×60-×100 oil immersion objective using an Olympus BX-61 fluorescence microscope (Center Valley, PA). A tumor was considered *ALK* rearrangement positive if more than 15% of 50 (minimum) or 100 analyzed tumor cells showed split probes signals or isolated orange signals in accordance with published IASLC guidelines (IASLC Atlas of *ALK* Testing in Lung Cancer).

## Results

### Establishment of the targeted NGS platform to detect DNA alteration using DNA samples and cancer cell lines

**Fig. 1:**
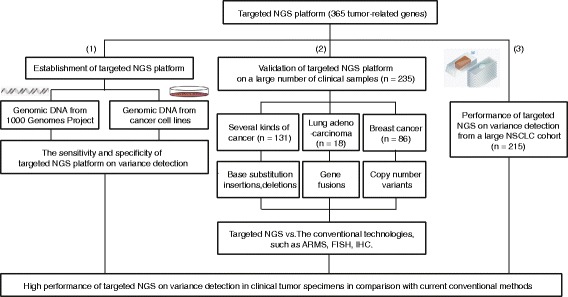
The summary of this study. First step:The genomic DNA from 1000 Genomes Project and the genomic DNA from cancer cells were used to set up basic parameters of targeted NGS platform; The second step: The validation of targeted NGS platform was performed on a large number of clinical samples (*n* = 235); The third step: Performance of targeted NGS on variance detection from a large NSCLC cohort (*n* = 215). In brief, these results suggested high performance of targeted NGS on variance detection in clinical tumor specimens

To establish a targeted NGS platform, we designed 16,198 DNA probes, targeting 4557 exons of 365 cancer-related genes, 47 introns of 25 genes frequently re-arranged in cancers. The capabality of the platform on DNA alteration detection was first tested in DNA samples and cancer cell lines (Fig. 1).

In DNA samples, we created DNA pools of normal cell lines from the 1000 Genomes Project, containning thousands of single nucleotide polymorphisms (SNPs) across the targeted exons that spans a broad range of MAF (5–100%) (Additional files 5 and 6: Table S5 and S6). On the other hand, 10 cancer cell lines haboring known somatic base substitutions and Indels were collected for this study. Two to ten of these 10 cell lines were randomly chosen and mixed with equal amount of DNA to form 21 pools. These 21 pools were sequenced by the targeted NGS, and the minimum sequencing coverage is 811× (Additional file 7: Table S7). 548 sites for base substitutions and 65 sites for Indels were selected for analysis across 21 pools (Additional files 8 and 9: Table S8 and S9).

**Fig. 2:**
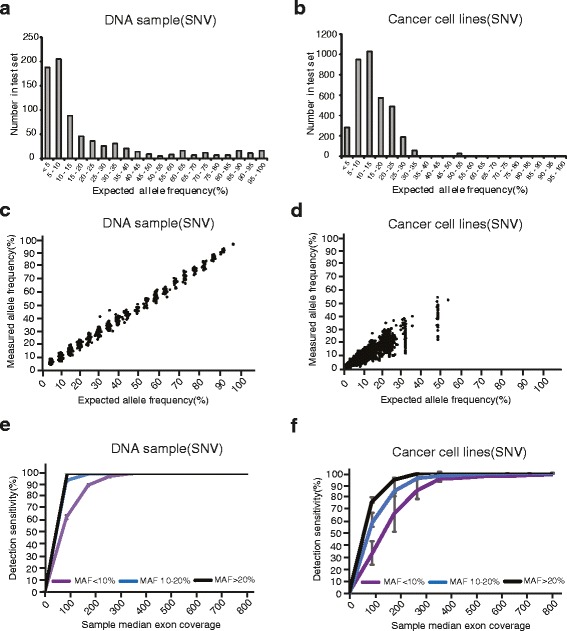
Establishment of the targeted NGS platform to detect DNA alteration using DNA samples and cancer cell lines. The reference standard DNA samples and the genomic DNA from cancer cells were sequenced by the targeted NGS. The distribution of detected SNV by mutation allele frequencies (MAFs) was illustrated in (**a**) and (**b**). The scatter plots in (**c**) and (**d**) represent the consistancy between measured MAFs and expected MAFs. The sensitivities of SNV detection were shown in (**e**) and (**f**). Error bars, s.e.m.

In standard DNA pools, 97.5% base substitutions’ MAF in cancer cell line pools are less than 30%, while 72.3% base substitutions’ MAF in DNA samples are less than 30% (Fig. 2a and b). The MAFs calculated from targeted NGS were highly consistent with the expected ones in normal DNA pools (Fig. 2c). However, the MAFs of base substitutions in cancer cell line pools determined by targeted NGS are less correlated with the expected ones (Fig. 2d), which may be due to non-diploid genomes in cancer cells. The complexity of the cancer cell lines themselves may better represent the real world clinical samples. Therefore cancer cell lines instead of the normal cell DNA could be better for the NGS platform validation.

To test the correlation between sensitivity and sequencing depth, the sequencing reads were randomly selected to in silico form a set of fastq files with sequencing depth from 0× to 800×. As expected, detection sensitivity declined with decrease of coverages, especially for those base substitutions with a MAF lower than 10% (Fig. 2e and f). The average detection sensitivities reach plateau at the coverage of 400× for base substitutions in normal DNA samples and at 500× in cancer cell line pools (Fig. 2e and f). For base substitutions with a MAF of ≤10%, 10–20% and ≥20%, the sensitivities at 500× coverage in cancer cell lines were 97.3% (1200/1233), 99.7% (1648/1653) and 100% (719/719), respectively (Fig. 2f). These data demonstrate that the targeted NGS has high sensitivity on detection of base substitutions.

For the base substitutions with a MAF of ≤10%, 10–20% and ≥20%, the high sensitivities of detection are reached in both DNA samples and cancer cell lines at relative high coverage, while the speed of reaching the maximum in cancer cell lines is much slower than the one in DNA samples (Fig. 2e and f). Furthermore, the variances at different MAF groups in caner cell lines are larger than the ones in DNA samples. In other words, when the sequence depth is low, all of the real mutations can not be detected in cancer cell lines. It is suggested that the cancer cell lines can better represent the complicated features of tumor heterogeneity, and may be better for NGS platform validation.

To assess the capacity of the targeted NGS on detection of Indels, the total 1365 (MAF ≥ 1%) Indels were known in cancer cell lines and most MAF is less than 30% (Additional file 10: Fig. S1a). At 400×, the sensitivity can reach to 97.8%. Due to the complexity of the cancer cell lines, the MAFs of indels in cancer cell lines determined by targeted NGS are less correlated with the expected ones (Additional file 10: Fig. S1b). Overall, the targeted NGS has high sensitivity on detection of Indels with the relatively low sequencing depth compared with the base substitutions (Additional file 10: Fig. S1c).

To test the capacity of the targeted NGS on detection of CNV, DNA from the cell line HCC1143 with known amplifications of *CCND1*, *FGF3*, *FGF4*, *FGF19* and *AKT1*, was diluted with matched normal DNA from 50% to 10%. At 50%, all known amplifications were detected, but at 20%, *AKT1*-amplification was undetectable (Additional file 10: Fig. S1d and S1e). It is concluded that more than 20% tumor cells in the mixed cell line pool are required to reach a high sensitivity of CNV detection. This is used as the guidline for a clinical test.

### Targeted NGS to identify base substitutions and Indels from clinical specimens

**Fig. 3:**
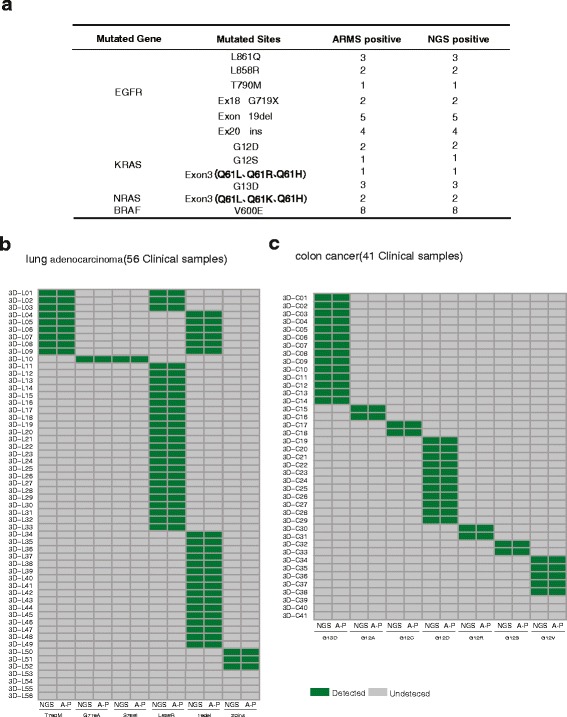
Targeted NGS to identify base substitutions and Indels from clinical specimens. Mutations detected by targeted NGS and ARMS-PCR in 34 FFPE resection specimens, 56 lung adenocarcinoma specimens, and 41 colorectal cancer specimens were illustrated in (**a**), (**b**), and (**c**), respectively

To evaluate the capacity of targeted NGS to detect base substitutions and Indels from clinical specimens, we collected 34 FFPE resection specimens including 17 lung cancers, 13 colorectal cancers and 4 melanomas. Total of 12 mutation sites in four oncogenes (*EGFR*, *KRAS*, *NRAS* and *BRAF*) had been identified previously by ARMS in the hospital. Each specimen harbors at least one DNA aberration. The DNA alterations in these samples were then analyzed by targeted NGS. As was shown in Fig. 3a and Additional file 11: Table S10, all DNA aberrations were also detected by targeted NGS, indicating that the targeted NGS is as sensitive as ARMS to identify base substitutions and indels.

To further compare the detection efficiency between targeted NGS and ARMS, we gathered another batch of FFPE resection specimens, including 56 lung adenocarcinoma and 41 colorectal cancers. These specimens had been examined by targeted NGS, and 15 hotspot mutation sites in *EGFR* and *KRAS* (52 *EGFR*-mutated specimens,38 *KRAS*-mutated specimens and 7 specimens without these hot mutated sites) (Additional file 10: Fig. S2) were identified. Because of the complexity of variant in clinical tumor samples, there are a multiple of genetic muations in one tumor sample (Additional file 10: Fig. S2). Among these hotspot mutations from 97 specimens, all samples were confirmed by ARMS, and the concordance is 100% at the sample level (Fig. 3b, c and Additional file 12: Table S11). Overall, targeted NGS is as sensitive as ARMS to detect hotspot base substitutions and Indels from clinical FFPE samples.

### Targeted NGS to detect DNA rearrangements from clincal specimens

**Fig. 4:**
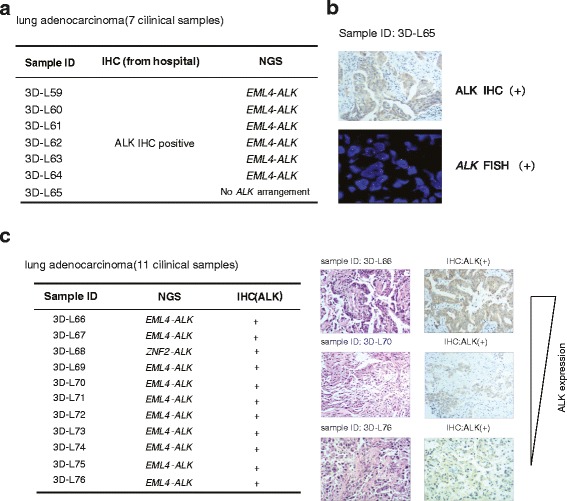
Targeted NGS to detect DNA rearrangements from clincal specimens. (**a**) 7 FFPE resection specimens with *ALK* fusions identified by IHC were further analyzed by targeted NGS. The results were shown on the table. *ALK* fusion in sample 3D-65 was further confirmed by FISH (**b**). (**c**) 11 lung adenocarcinoma FFPE specimens that are positive in *ALK* fusions by targeted NGS were further analyzed by IHC. The results were summarized in the left table. ‘+’ represents positive ALK expression detected by IHC. Representative microscopical results of ALK expression from high to low are shown on the right panel

We collected 18 FFPE resection specimens of lung adenocarcinoma to assess the capacity of targeted NGS to detect DNA arrangements. Among these 18 samples, 7 resection specimens had been stained by IHC and showed positive ALK immunostaining, indicating *ALK* fusions (Fig. 4a). These samples were re-examined by targeted NGS. The data revealed that 6 samples possessed *EML4*-*ALK* fusions, while 3D–L65 showed negative *ALK* fusion (Fig. 4a). Sample 3D–L65 was further investigated by IHC and FISH from the third party and confirmed to be *ALK* positive (Fig. 4b). The inconsistency with the targeted NGS’s result could be contributed by the tumor heterogeneity or no probe coverage due to new fusion types. The remaining 11 FFPE samples were examined by targeted NGS and were positive *ALK* rearrangements (Fig. 4c). Among these variants of *ALK* fusions in this study, *ZNF2*-*ALK* had not been reported previously. All these samples were then immunostained with ALK antibody, and were identified to be ALK positive. The specificity of *ALK* fusion identification from targeted NGS is 100% (Fig. 4c). Therefore, targeted NGS is sensitive enough to identify *ALK* fusion across a large range of ALK expression level, which demonstrates high tumor heterogeneity.

### Targeted NGS to identify CNVs from clinical specimens

**Fig. 5:**
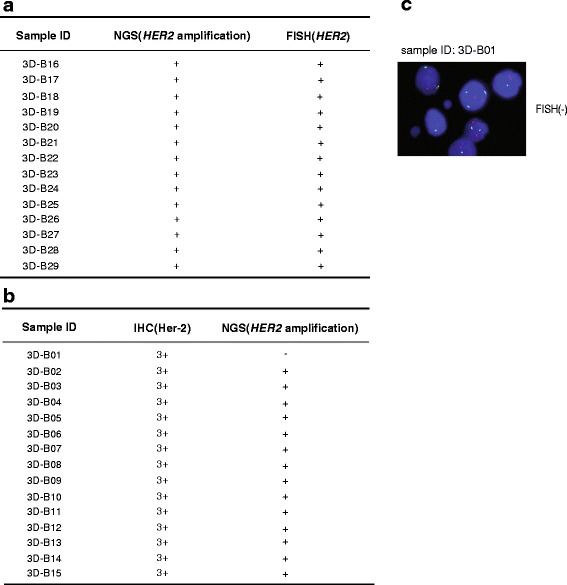
Targeted NGS to identify CNVs from clinical specimens. (**a**) The 14 breast cancer samples with NGS (*HER2* amplification) positive were confirmed by FISH. (**b**) The results of 15 breast cancer samples with IHC (Her-2) 3+ were detected by targeted NGS. (**c**) The discordant result from sample 3D–B01 was further confirmed by FISH

*HER2* is frequently amplified in breast cancers. [12] FISH is recognized as the “gold standard” for translocations and *HER2* amplification. To investigate the detection efficiency of targeted NGS on CNVs, FISH is used to confirm HER2-amplification detected by targeted NGS. In 14 samples with the positive *HER2* amplification by targeted NGS, all samples were also positively confirmed by FISH (Fig. 5a). In comparison with the golden standerd FISH, the specificity of targeted NGS was 100% (14/14).

Since IHC is a widely used clinical method in China, and most breast specimens were immunostained with Her-2 antibody. The staining intensities were classified into 5 groups ranging from low to high: -,1+, 2+, 2 + ~3+ and 3+, which demonstrated that clinical breast cancer samples was too complicated to be divided into positive and negtive groups. Among the 15 *HER2*-overexpressed samples (IHC 3+), 14 were identified to be *HER2* amplification by targeted NGS (Fig. 5b). The disconcordant case, 3D–B01, was further identified to be no *HER2* amplification by FISH (Fig. 5c), which suggests that overexpression of Her-2 protein in this case was not contributed by *HER2* amplification. All 35 Her-2 negative (−, +) specimens were no *HER2* amplification by targeted NSG (Additional file 10: Fig. S3a). In comparison with IHC 3+ results, the sensitivity and specificity of targeted NGS were 93.3% (14/15) and 100% (35/35), respectively (Fig. 5b and Additional file 10: Fig. S3a). For the specimens with the IHC transient state (2+ and 2 + ~3+), the concordance between IHC and targeted NGS is less than 50% (Additional file 10: Fig. S3a). It is suggested that high Her-2 protein expression is not only contributed by *HER2* amplification.

To further elucidate the specificity of CNV identification by NGS, eight samples from multiple types of cancers have been identified to be *HER2* amplification by targeted NGS. All of them have been stained by IHC and 7 out of 8 samples were scored 3+ and one scored 2+. This suggested that *HER2* amplification detected by NGS leads to high Her-2 protein expression (Additional file 10: Fig. S3b).

### Performance of targeted NGS on variance detection from a large non-small-cell lung cancer (NSCLC) cohort

**Fig. 6:**
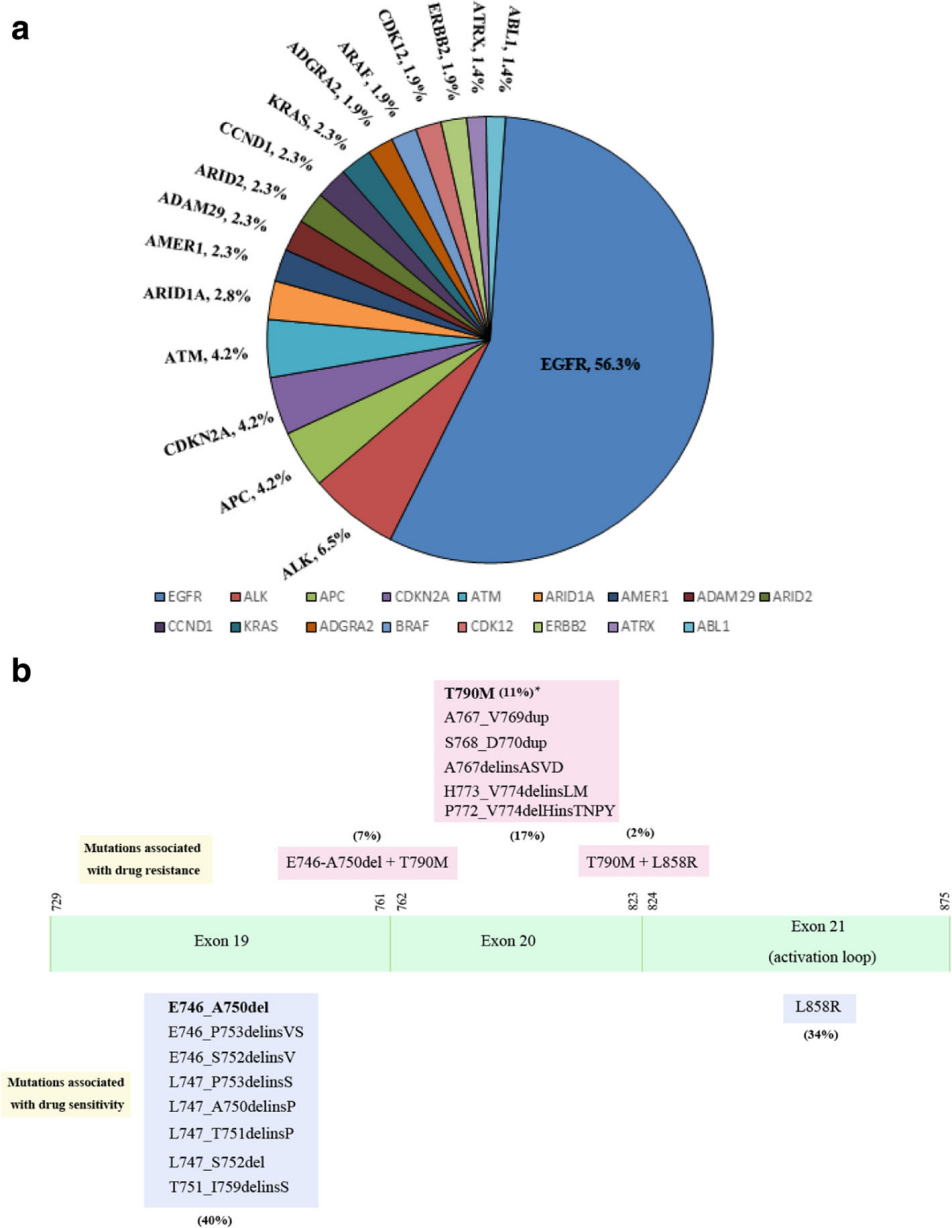
Targeted NGS futher validated based on the spectrum of DNA alterations and EGFR hotspot mutation rates in a large non-small-cell lung cancer (NSCLC) cohort. (**a**) Gene alterations were found in 215 cases of NSCLC by targeted NGS. (**b**) Hotspot mutations in *EGFR* were identified by targeted NGS platform in 121 NSCLC samples

To further prove the reliability of the targeted NGS platform on DNA alteration detection, we analyzed the spectrum of DNA alterations identified by the platform. In 215 NSCLS cases, 17 genes were identified to have the multiple types of variants (Additional file 13: Table S12). Most of them are known NSCLS driver genes, such *EGFR*, *CDKN2A*, *ALK* and etc. (Fig. 6a). The recurrent frequency of *EGFR* mutations was 56.3%, very close to the reported results in NSCLC patients in China, 46.6% ~ 53.8%. [10] The other oncogenes also had similiar recurrent frequencies as reported in TCGA (The Cancer Genome Atlas) and other studies. [13, 14]

In order to investigate the detection of targeted NGS on *EGFR* hotspot mutations, we analyzed 121 NSCLC samples with *EGFR* mutations detected by targeted NGS (Additional file 14: Table S13). In these samples, the *EGFR* mutations associated with drug sensitivity, such as L858R and some 19 exon deletions (or insertions), were detected by targeted NGS. Moreover, the mutation rates of L858R and 19 exon deletions (or insertions) were 34% and 40% respectively (Fig. 6b), and were similar to that from other studies. [15–17] In addition, the *EGFR* mutations associated with drug resistance, such as T790 M and 20 exon mutations, [18–20] were also found in these NSCLC samples. In conclusion, the targeted NGS platform developed here has accurately detect *EGFR* hotspot mutations in NSCLC patients.

## Discussion

We established a NGS platform targeting 365 cancer-related genes to identify genomic DNA alterations including base substitutions, Indels, rearrangement and CNV. In this study, we have answered to the question – “what kind procedures need to throughly validate the new NGS platform for clinical diagonasis”. This newly established NGS platform has been compared with the current clinical platforms, such as ARMS, IHC and FISH in the detection of different types of variances. Although high concordance exists between the platforms, the minor difference does demonstrate the uniqueness for each platform. For the targeted NGS, one assay can identify mutiple types of variances. The disvantage is that the turn around time (TAT) is too long for the clinical diagnosis in comparison with ARMS, IHC and FISH. If the clinical purpose is clear, the current clinical tool would be better in term of TAT and cost. This provides a clinician with the challenge to choose the right platform.

For the known hotspot variance detection like *EGFR* L858R and 19dels, the high concordence demonstrates that targeted NGS is as efficient as ARMS does. For those patients who do not have the hotspot mutations covered by ARMS, targeted NGS may identify the new mutations due to unbias probe design in all interesting regions, which can provide the hope for a new treatment. Specially for the relapse patient, he or she has developed resistance to tyrosine kinase inhibitors (TKIs).

*ALK* rearrangement generates an oncogenic fusion kinase leading to ALK constitutive activation. [21, 22] *ALK* rearrangement occurs in around 3–6% NSCLC, and is a promising therapeutic target. [23] An ALK inhibitor like crizotinib has benefit the lung adenocarcinomas patient with *ALK* rearrangement. [24] FISH is the only diagnostic tool approved by Food and Drug Administration (FDA) to identify *ALK* rearrangement. Although FISH has a high sensitivity and specificity, it can not distinguish all *ALK* fusion types, which are associated with the efficacy of crizotinib in patients. [25] Several studies reported that a very high concordance between IHC and FISH exists, [26, 27] and IHC to determine *ALK* status was also approved by China Food and Drug Administration (CFDA). However, ALK antibody affinity to its fusion proteins may depend on specific variances. For example, ALK antibody CD246 from Dako only has 27% sensitivity to *EML4*-*ALK* variances 1 and 3a/b. [28] Moreover, intracellular and extracellular mucin have effect on IHC analysis, which may cause high false-negative and false-positive detection respectively. [29] Although IHC and FISH have some disvantages, they can detect some types of *ALK* rearrangements that targeted NGS can not identify due to the complexity of clinical specimens. While targeted NGS is as efficient as IHC on *ALK* fusion detection and avoides IHC above weakness, it can also identify new *ALK* rearrangement.

As to *HER2* amplifications, IHC is not as effective as FISH for the detection of *HER2* amplification because IHC is mainly for protein expression. Protein expression level is highly correlated with amplification, but not one to one relationship exists. In this study, Her-2 IHC 3+ was highly concordant with FISH results, but IHC 3 + −2+ or 2+ showed a large discordance with the FISH results. [30] FISH has a higher predictive value than IHC for response to treatment with trastuzumab which targets Her-2. [31] Our study demonstrated that the concordance of targeted NGS in the detection of *HER2* amplification was 100%, in comparison with the golden standern FISH. In light of CNV detection, targeted NGS has advantage over IHC.

## Conclusions

In conclusion, our study disclosed that DNA from cancer cell lines is better than standard DNA as a reference to establish basic parameters for targeted NGS. In spite of the complexity of clinical specimens, comparison of the conventional methods using a large cohort of patient samples confirmed that targeted NGS has relatively high performance to identify multiple genomic alterations in a single assay. But the throughly validation of the new platform for clinical diagnosis is necessary and highly recommended.

## Additional files

Additional file 1: Table S1. The information of 235 tumour specimens. (XLS 40 kb)

Additional file 2: Table S2. DNA sample information. (XLS 24 kb)

Additional file 3: Table S3. The list of immortalized tumor cell lines and pools. (XLS 26 kb)

Additional file 4: Table S4. The gene list of targeted NGS platform. (XLSX 12 kb)

Additional file 5: Table S5. The golden standard sites of SNP in DNA sample_pool1. (XLS 589 kb)

Additional file 6: The golden standard sites of SNP in DNA sample_pool2. (XLS 594 kb)

Additional file 7: Table S7. Quality control results of samples detected by NGS. (XLS 34 kb)

Additional file 8: Table S8. The golden standard sites of SNP in cancer cell line validation. (XLS 68 kb)

Additional file 9: Table S9. The golden standard sites of Indel in cancer cell line validation. (XLS 27 kb)

Additional file 10: Supplementary Figures S1-S3. **Figure S1.** The results of Indels and CNVs detected by the means of targeted NGS in cancer cell lines; **Figure S2.** Targeted NGS comparable to ARMS in Lung adenocarcinoma and Colon Cancer FFPE samples; **Figure S3.** The comparation between targeted NGS and IHC on the HER2 amplification in clinical FFPE samples. (DOCX 572 kb)

Additional file 11: Table S10. The comparation of ARMS and targeted NGS in 34 clinical samples. (XLS 29 kb)

Additional file 12: Table S11. The comparation of targeted NGS and ARMS in 97 clinical samples. (XLS 45 kb)

Additional file 13: Table S12. The spectrum of DNA alternations in non-small-cell lung cancer. (XLS 25 kb)

Additional file 14: Table S13. EGFR hotspot mutations identified by targeted NGS in NSCLC. (XLS 34 kb)

## Abbreviations

ARMS: amplification-refractory mutation system
ATCC: American Type Culture Collection
CFDA: China Food and Drug Administration
CNV: copy number variance
FDA: Food and Drug Administration
FFPE: formalin fixed, paraffin-embedded
FISH: fluorescence in situ hybridization
IHC: immunohistochemistry
MAF: mutation allele frequency
NGS: Next generation sequencing
NSCLC: non-small-cell lung cancer
SNP: single nucleotide polymorphisms
SNVs: single nucletic variances
TAT: turn around time
TCGA: The Cancer Genome Atlas
TKI: tyrosine kinase inhibitor

## References

[1] Lopez JS, Banerji U (2017). Combine and conquer: challenges for targeted therapy combinations in early phase trials. Nature reviews. Clin Oncol.

[2] Riely GJ, Pao W, Combining EGFR (2005). Targeted therapy with chemotherapy in pancreatic cancer: is timing important?. Cancer Biol Ther.

[3] Stewart EL, Tan SZ, Liu G, Tsao MS (2015). Known and putative mechanisms of resistance to EGFR targeted therapies in NSCLC patients with EGFR mutations-a review. Transl Lung Cancer Res.

[4] Diaz LA, Jr., Bardelli A. Liquid biopsies: genotyping circulating tumor DNA. J Clin Oncol 2014;32(6):579–586. 10.1200/JCO.2012.45.2011 PMC4820760 24449238

[5] Frampton GM, Fichtenholtz A, Otto GA, Wang K, Downing SR, He J (2013). Development and validation of a clinical cancer genomic profiling test based on massively parallel DNA sequencing. Nat Biotechnol.

[6] Lefebvre C, Rieckhof G, Califano A (2012). Reverse-engineering human regulatory networks. Wiley Interdiscip Rev Syst Biol Med.

[7] Shrager J, Tenenbaum JM (2014). Rapid learning for precision oncology. Nat Rev Clin Oncol.

[8] Fisher S, Barry A, Abreu J, Minie B, Nolan J, Delorey TM (2011). A scalable, fully automated process for construction of sequence-ready human exome targeted capture libraries. Genome Biol.

[9] Karolchik D, Hinrichs AS, Furey TS, Roskin KM, Sugnet CW, Haussler D (2004). The UCSC table browser data retrieval tool. Nucleic Acids Res.

[10] Shi Y, JS A, Thongprasert S, Srinivasan S, Tsai CM, Khoa MT (2014). A prospective, molecular epidemiology study of EGFR mutations in Asian patients with advanced non-small-cell lung cancer of adenocarcinoma histology (PIONEER). J Thorac Oncol.

[11] McLeer-Florin A, Moro-Sibilot D, Melis A, Salameire D, Lefebvre C, Ceccaldi F (2012). Dual IHC and FISH testing for ALK gene rearrangement in lung adenocarcinomas in a routine practice: a French study. Journal of thoracic oncology : official publication of the International Association for the Study of Lung Cancer.

[12] Slamon DJ, Godolphin W, Jones LA, Holt JA, Wong SG, Keith DE (1989). Studies of the HER-2/neu proto-oncogene in human breast and ovarian cancer. Science.

[13] Pao W, Girard N (2011). New driver mutations in non-small-cell lung cancer. Lancet Oncol..

[14] Swanton C, Govindan R (2016). Clinical implications of genomic discoveries in lung cancer. N Engl J Med.

[15] Pirker R, Herth FJ, Kerr KM, Filipits M, Taron M, Gandara D (2010). Consensus for EGFR mutation testing in non-small cell lung cancer. J Thorac Oncol.

[16] Pao W, Chmielecki J (2010). Rational, biologically based treatment of EGFR-mutant non-small-cell lung cancer. Nat Rev Cancer.

[17] Mitsudomi T, Yatabe Y (2007). Mutations of the epidermal growth factor receptor gene and related genes as determinants of epidermal growth factor receptor tyrosine kinase inhibitors sensitivity in lung cancer. Cancer Sci.

[18] Chen D, Song Z, Cheng G (2016). Clinical efficacy of first-generation EGFR-TKIs in patients with advanced non-small-cell lung cancer harboring EGFR exon 20 mutations. Onco Targets Ther.

[19] Naidoo J, Sima CS, Rodriguez K, Busby N, Nafa K, Ladanyi M (2015). Epidermal growth factor receptor exon 20 insertions in advanced lung adenocarcinomas: clinical outcomes and response to erlotinib. Cancer.

[20] Greulich H, Chen TH, Feng W, Janne PA, Alvarez JV, Zappaterra M (2005). Oncogenic transformation by inhibitor-sensitive and -resistant EGFR mutants. PLoS Med.

[21] Soda M, Choi YL, Enomoto M, Takada S, Yamashita Y, Ishikawa S (2007). Identification of the transforming EML4-ALK fusion gene in non-small-cell lung cancer. Nature.

[22] Rikova K, Guo A, Zeng Q, Possemato A, Yu J, Haack H (2007). Global survey of phosphotyrosine signaling identifies oncogenic kinases in lung cancer. Cell.

[23] Shaw AT, Yeap BY, Solomon BJ, Riely GJ, Gainor J, Engelman JA (2011). Effect of crizotinib on overall survival in patients with advanced non-small-cell lung cancer harbouring ALK gene rearrangement: a retrospective analysis. Lancet Oncol.

[24] Shaw AT, Kim DW, Nakagawa K, Seto T, Crino L, Ahn MJ (2013). Crizotinib versus chemotherapy in advanced ALK-positive lung cancer. N Engl J Med.

[25] Yoshida T, Oya Y, Tanaka K, Shimizu J, Horio Y, Kuroda H (2016). Differential Crizotinib response duration among ALK fusion variants in ALK-positive non-small-cell lung cancer. Journal of clinical oncology : official journal of the American Society of Clinical Oncology.

[26] Cabillic F, Gros A, Dugay F, Begueret H, Mesturoux L, Chiforeanu DC (2014). Parallel FISH and immunohistochemical studies of ALK status in 3244 non-small-cell lung cancers reveal major discordances. J Thorac Oncol.

[27] Ali G, Proietti A, Pelliccioni S, Niccoli C, Lupi C, Sensi E (2014). ALK rearrangement in a large series of consecutive non-small cell lung cancers: comparison between a new immunohistochemical approach and fluorescence in situ hybridization for the screening of patients eligible for crizotinib treatment. Arch Pathol Lab Med..

[28] Wallander ML, Geiersbach KB, Tripp SR, Layfield LJ (2012). Comparison of reverse transcription-polymerase chain reaction, immunohistochemistry, and fluorescence in situ hybridization methodologies for detection of echinoderm microtubule-associated proteinlike 4-anaplastic lymphoma kinase fusion-positive non-small cell lung carcinoma: implications for optimal clinical testing. Arch Pathol Lab Med.

[29] Yu Y, Ding Z, Zhu L, Teng H, Frequencies LS (2016). Of ALK rearrangements in lung adenocarcinoma subtypes: a study of 2299 Chinese cases. Spring.

[30] Wesola M, Jelen MA (2015). Comparison of IHC and FISH cytogenetic methods in the evaluation of HER2 status in breast cancer. Adv Clin Exp Med.

[31] Pauletti G, Dandekar S, Rong H, Ramos L, Peng H, Seshadri R (2000). Assessment of methods for tissue-based detection of the HER-2/neu alteration in human breast cancer: a direct comparison of fluorescence in situ hybridization and immunohistochemistry. J Clin Oncol.

