# Supplementary material for: High performance of targeted next generation sequencing on variance detection in clinical tumor specimens in comparison with current conventional methods
Source: J Exp Clin Cancer Res. 2017 Sep 7;36:121. doi: 10.1186/s13046-017-0591-4 (PMC5590190; doi:10.1186/s13046-017-0591-4)
Supplement: Supplementary file 10 — Figure S1. The results of Indels and CNVs detected by the means of targeted NGS in cancer cell lines; Figure S2. Targeted NGS comparable to ARMS in Lung adenocarcinoma and Colon Cancer FFPE samples; Figure S3. The comparation between targeted NGS and IHC on the HER2 amplification in clinical FFPE samples. (DOCX 572 kb) [file 13046_2017_591_MOESM10_ESM.docx]

**List of all Supplemental Items**

**Figure S1,** **Related to Figure 1.**

**Figure S2, Related to Figure 2.**

**Figure S3, Related to Figure 4.**


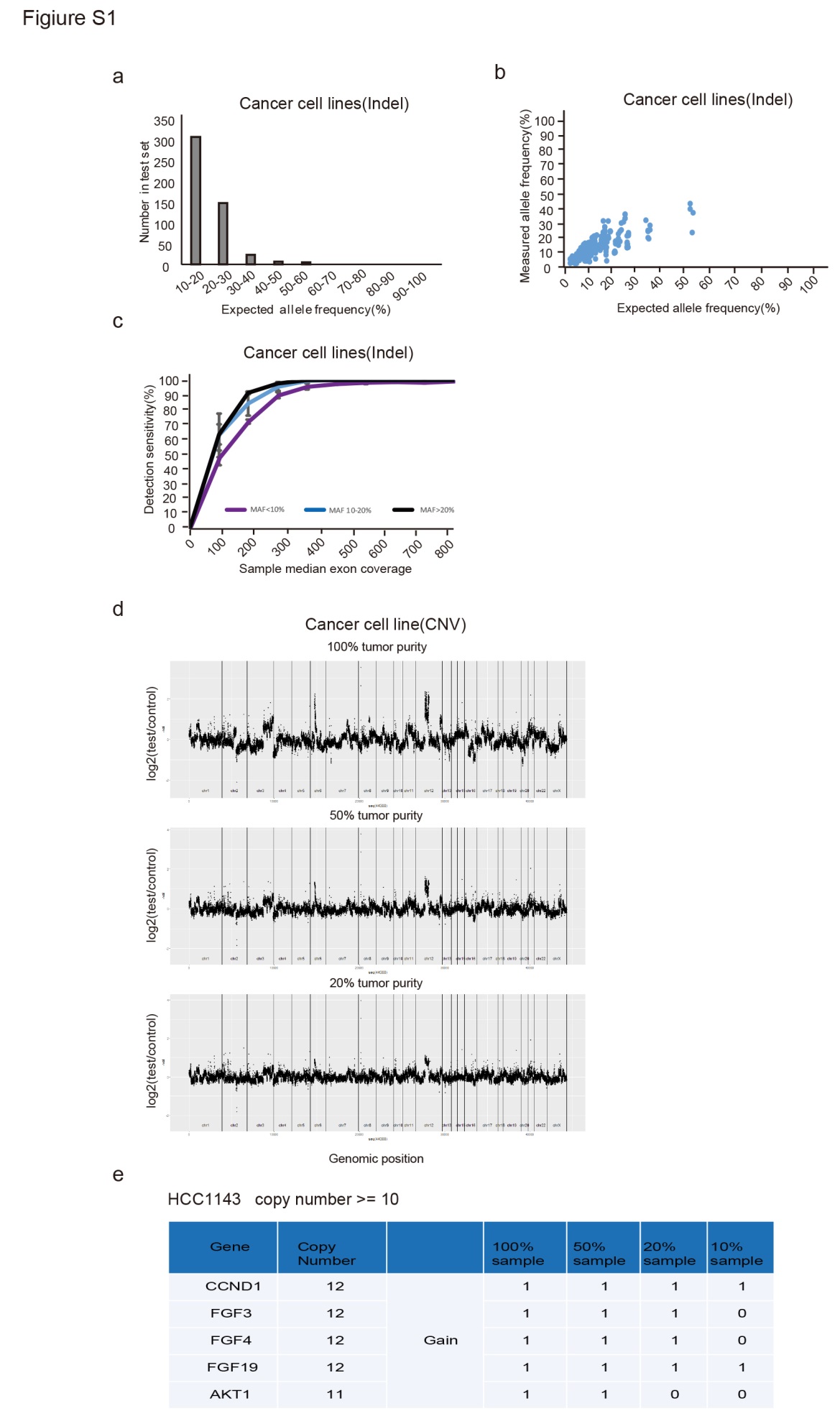


**Figure S1: The results of Indels and CNVs detected by the means of targeted NGS in cancer cell lines**

The DNA pools obtained by mixing DNAs from 21 cancer cell lines were sequenced by the targeted NGS to determine the MAFs and numbers of base substitutions (A), as well as the MAFs and expected allelle frequency detection of Indels (B). Detection sensitivity of Indels as a function of sample median exon coverage in cancer cell lines (C). Error bars, s.e.m. (D) DNA from cell line HCC1143 mixed with matched DNA from normal cells at a ratio of 100%, 50% or 20%, and then sequenced by the targeted NGS, followed by CNV analysis. Y axes denote log-ratio measurements of coverage obtained in mixture samples versus the normal sample. X axes represent the genomic positions. (E) Summary of copy number alteration detection performance in HCC1143 (copy number >= 10). ‘1’ represents CNVs detected, while ‘0’ represents CNVs undetected.


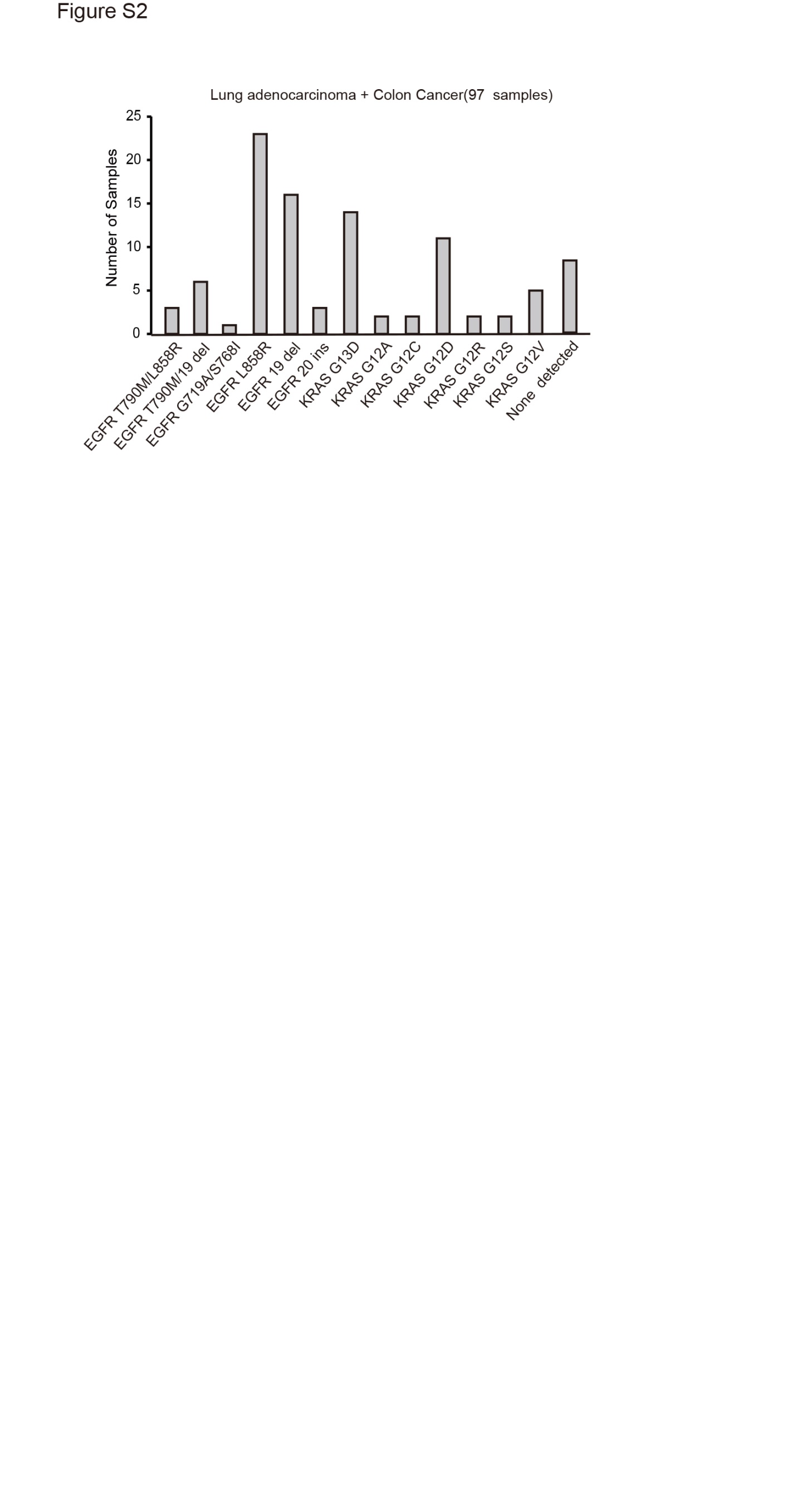


**Figure S2: Targeted NGS comparable to ARMS in Lung adenocarcinoma and Colon Cancer FFPE samples**

The types and numbers of DNA alternations in the 97 specimens were shown.

**
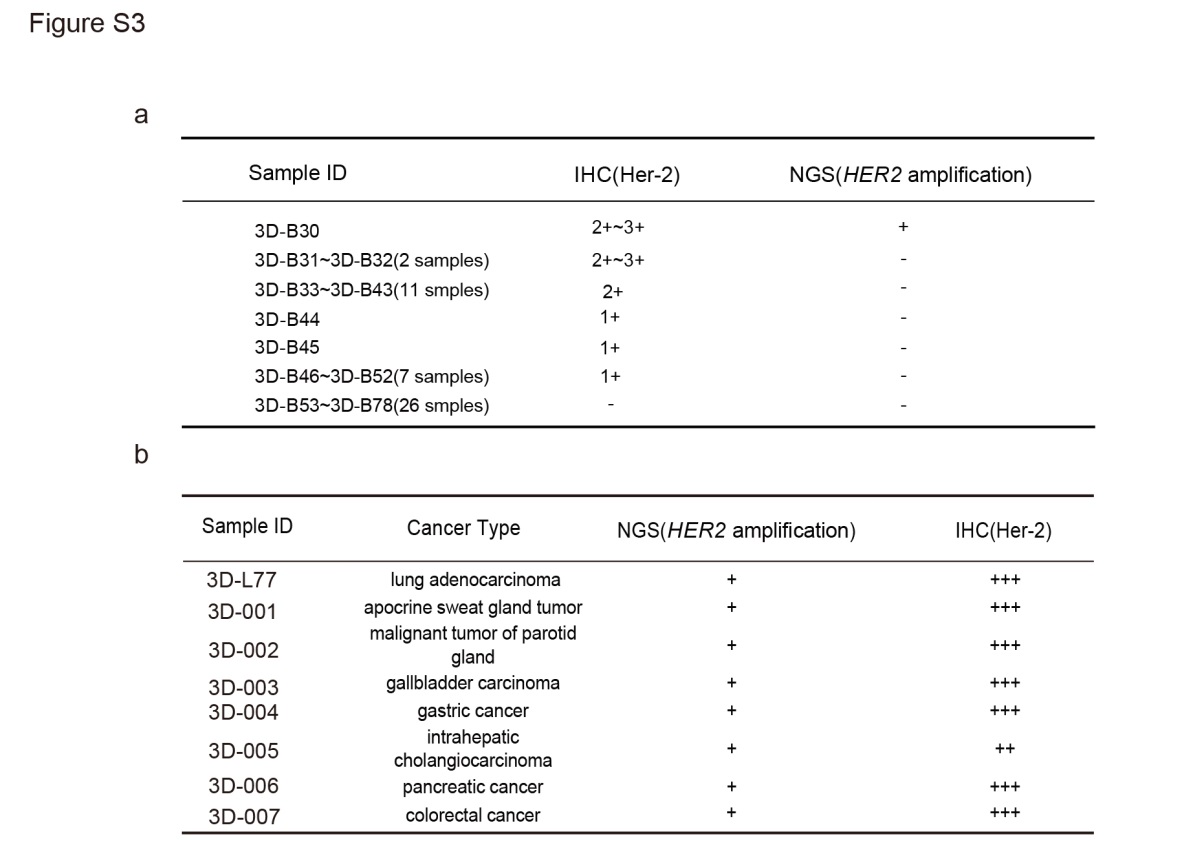
**

**Figure S3: The comparation between targeted NGS and IHC on the HER2 amplification in clinical FFPE samples**

(A) The results of breast cancer samples with Her-2 expression level(2+~3+, 2+,1+,-) were detected by targeted NGS.(B) The results of 8 other caner specimens (lung adenocarcinoma, gallbladder carcinoma, gastric cancer, intrahepatic cholangiocarcinoma, apocrine sweat gland tumor, malignant tumor of parotid gland, colorectal cancer, pancreatic cancer) were detected by targeted NGSand IHC test.
